# A Retrospective, Digital Evaluation of Tip and Torque of Teeth in Patients with Skeletal Class I, II and III Using Lateral Cephalograms, Orthopantomograms and Digitized Models

**DOI:** 10.3390/jcm14217738

**Published:** 2025-10-31

**Authors:** Corinna L. Seidel, Karolina Kelemenova, Uwe Baumert, Andrea Wichelhaus, Hisham Sabbagh

**Affiliations:** Department of Orthodontics and Dentofacial Orthopedics, University Hospital, LMU Munich, Goethestrasse 70, 80366 Munich, Germany; karolina.kelemenova@med.uni-muenchen.de (K.K.); uwe.baumert@med.uni-muenchen.de (U.B.); kfo.sekretariat@med.uni-muenchen.de (A.W.); hisham.sabbagh@med.uni-muenchen.de (H.S.)

**Keywords:** orthodontics, models, dental, radiography, panoramic, radiography, dental, digital, dental occlusion

## Abstract

**Objectives**: Knowledge of tooth axes is important in orthodontics; however, using just one method for evaluation, e.g., orthopantomograms for tip, is not highly reliable. This study aimed to investigate tooth axes in skeletal class I/II/III using two- and three-dimensional evaluations. **Methods**: In this retrospective study, lateral cephalometric radiographs, orthopantomograms and digitized models of 107 adolescent patients (*Ø* 13.5 years; *n* = 36/33/38 with cI/cII/cIII) prior to orthodontic treatment were analyzed digitally regarding tip and torque of teeth. Statistical analysis was performed using SPSS (*p* ≤ 0.05), G*power and a multiple testing tool (Bonferroni–Holm/Hochberg). **Results**: Dental compensation of skeletal cII/cIII was significant acc. to Bonferroni–Holm/Hochberg for the following variables: overjet compensation in cII was seen by more retroinclined upper incisors in cII by −5.9°/−5.3° and by −8.8°/−6.6° (U1-SN/U1-PP) vs. cI/cIII (effect size *f* = 0.489/0.446, power 0.996/0.988). In cIII, the lower incisors were more retroinclined by −8.5°/−10.9° (L1-MP) vs. cI/cII (*f* = 0.576, power 1.000) and by −8.5°/−8.9° and −6.0°/−7.0° (three-dimensional analysis: L1/L2) vs. cI/cII (*f* = 0.522/0.527, power 0.999). Compensation of distal occlusion was found by mesial tipping of L3 by 3.5° in cII (*f* = 0.242, power 0.591) vs. cIII. CIII showed transversal compensation by buccal tipping of the U5 by 5.9°/4.6° vs. cII/I (*f* = 0.355, power 0.910) and lingual tipping of L3 by −6.4° vs. cII and −3.8° vs. cI (*f* = 0.446, power 0.988) and L4 by −4.0°/−2.6° vs. cII/I (*f* = 0.326, power 0.846). **Conclusions**: Decompensation, e.g., uprighting of distal tipped canines, and further protrusion of incisors might not be desired in orthodontic treatment of adolescents.

## 1. Introduction

While dental class I is the most prevalent occlusion, the prevalence of skeletal class II is higher than skeletal class I [1,2]. The higher prevalence of dental class I can be explained by the pursuit of harmonious occlusal relationships leading to dental compensation mechanisms in skeletal dysgnathia during the growth phase [2]. Regarding orthodontic treatment of patients with skeletal class II and III showing dental compensation, different treatment strategies can be indicated. When skeletal dysgnathia is severe and orthognathic surgery is required, decompensation of the dental compensation mechanism can be required in adult patients. For example, the decompensation of skeletal class III can require extraction of upper premolars [3] combined with increased anchorage in the upper jaw [4]. In some elderly patients, when skeletal class is less severe and facial profile is acceptable, dentoalveolar compensation (camouflage therapy) of skeletal dysgnathia might also be aimed at [5]. Regarding the orthodontic treatment of adolescent patients with permanent dentition, the maintenance of slight dental compensation of moderate skeletal class II and III can be indicated. Also, early treatment or functional orthodontic treatment during growth produce dental side effects next to the desired skeletal effects. For example, Sander-II appliance effectively reduces skeletal class II, while it also enhances retrusion of upper incisors as a dental compensation of an increased overjet [6]. Early treatment of skeletal class III, e.g., using a face mask for maxillary protraction, can lead to mesial migration of upper lateral teeth and protrusion of the upper incisors [7]. For daily practice, the knowledge of class-specific dental compensation mechanisms is necessary for biomechanical orthodontic treatment planning of adolescents. If orthodontic treatment is adolescents with skeletal class II or III is planned, the maintenance of either naturally occurring or phase I treatment-associated dental compensation patterns—to some extent—is necessary in most cases. Decompensation of dental compensation can aggravate the case and lead to the necessity of combined orthodontic–surgical treatment in adulthood.

With regard to the orthodontic treatment goal, Andrews [8] suggested that achieving a treatment result similar to that of untreated patients with naturally occurring ideal occlusion should be aimed at. In a study investigating plaster models of 120 Caucasian subjects with this ‘naturally optimal occlusion’, Andrews [8] evaluated—among other measurements—the tip and torque of the clinical crown of teeth. Based on these findings, the ‘six keys to optimal occlusion’ were defined, of which the second and third key present ‘ideal’ tips and inclinations of teeth [8]. However, several studies [9,10,11] detected differences compared to Andrews’ values in patients with ‘normal occlusion’. For example, most studies [12] used cone-beam computed tomography for investigation of tip and torque of the long axis and clinical crown in Chinese adult patients with ‘normal occlusion’. Also, studies have shown differences regarding tip and torque of teeth between patients with class I, II and III [13,14,15]; however, they did not apply more than one method and did not investigate all teeth: (i) Hernández-Sayago et al. [13] investigated lower incisor inclination using lateral cephalometric radiographs, (ii) Santana et al. [14] analyzed bucco-lingual torque of the first upper molars using digitized models, and (iii) Wang et al. [15] examined upper and lower incisor inclination using cone-beam computed tomography.

Hence, the aim of this study was to investigate tip and torque of teeth in adolescent patients with skeletal class I, II and III prior to orthodontic treatment using two- and three-dimensional analyses of digitized dental casts, lateral cephalometric radiographs and orthopantomograms. The overarching goal was to identify naturally occurring and/or pretreatment-associated dental compensation mechanisms in adolescent patients with skeletal class II and III regarding tooth axes to increase awareness in orthodontic treatment planning of those cases.

## 2. Materials and Methods

Prior to the beginning of the study, approval by the ethics committee of the University Hospital Munich, LMU Munich (vote number 20-1057, date of approval 18 December 2020), was attained. In this retrospective study, patients treated in the Department for Orthodontics and Orofacial Orthopedics, LMU Munich, between 1 January 2009 and 29 November 2020 were included. In total, 107 adolescent Caucasian patients (48 males, 59 females) were included in this study. As solely adolescent Caucasians were included, generalizability is limited as differences between ethnical groups regarding tooth axes were described [8,10,16,17,18,19]. Regarding skeletal class, 36 patients presented class I, 33 patients presented class II and 38 patients class III. The mean age was 13.5 ± 1.4 years (class I: 13.2 ± 1.0 years; class II: 13.1 ± 1.3 years; class III: 14.0 ± 1.6 years). The following inclusion criteria were applied: (i) fully toothed upper and lower jaw in permanent dentition (except second molars and wisdom teeth); (ii) complete orthodontic treatment records (orthopantomogram, lateral cephalometric radiographs, diagnostic plaster models, patient data including gender and age at the examination time point); (iii) classification to skeletal class I, II or III; (iv) time point prior to orthodontic treatment with fixed appliances; and (v) treatment with functional appliances before orthodontic treatment possible. Exclusion criteria were the following: (i) patients with syndromes; (ii) missing teeth (except wisdom teeth); (iii) displaced teeth; (iv) extraction cases; (v) patients requiring combined orthodontic and oral surgery treatments; (vi) dental implants; and (vii) diseases of the temporomandibular joint.

### 2.1. Cephalometric Measurements

All lateral cephalometric radiographs were taken with the ‘CS 9000 Extraoral Imaging System’ (Kodak, Rochester, NY, USA), and the diagnostic software ‘FR win professional’ (Computer Konkret AG, Falkenstein, Germany) was used to analyze the digital images. Cephalometric analyses were performed according to standard orthodontic cephalometrics [20,21,22,23] applied in the Department for Orthodontics of LMU Munich (Supplementary Table S1). It is recommended to consider several cephalometric parameters complementing each other for a more accurate assessment of skeletal dysgnathia [2,24]. Therefore, classification of skeletal class I, II and III was performed according to cephalometric measurements including the ANB angle [25] (skeletal class I: 0–4°, skeletal class II: >4°; skeletal class III: <0°), the individualized ANB [indiv.ANB = −35.16 + 0.4 × SNA(°) + 0.2 × ML-NSL(°)] [26] and Wits appraisal [27]. in accordance with Panagiotidis and Witt [26], the individualized ANB (indiv. ANB) was compared to the measured ANB angle for verification of the skeletal class in borderline cases. A difference of ±1° between the measured ANB value and the indiv. ANB indicated skeletal class I. If the measured ANB value deviated by more than 1° to the positive side from the indiv. ANB angle, skeletal class II was defined. If the actual ANB value deviated by more than 1° to the negative side from the indiv. ANB value, skeletal class III was defined.

### 2.2. Investigation of Tip of Teeth Using Orthopantomograms

**Figure 1:**
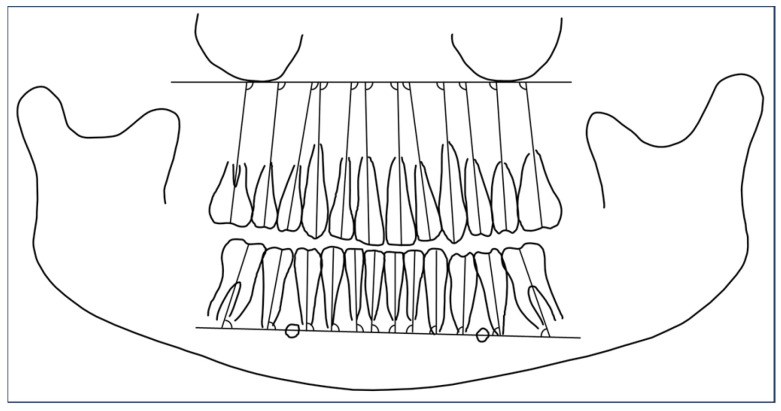
Graphical visualization of the orthopantomogram evaluation according to Ursi et al. [28].

All orthopantomograms were taken with the ‘CS 9000 Extraoral Imaging System’ (Kodak, Rochester, NY, USA) and analyzed using ‘FR win professional’ (version 07.00, Computer Konkret AG, Falkenstein, Germany). The mesio-distal tip of all teeth (except the second and third molars) were evaluated according to protocol introduced by Ursi et al. [28] (Figure 1).

First, anatomic landmarks like inferior lines of the orbits, mandible, foramen mentale and the outer contour of all teeth are drawn. The upper reference plane was traced between the most inferior points of orbits on both sides of the maxilla [28]. The lower reference line was plotted through the midpoint of the foramen mentale on both sides of mandibula [28]. The long axes of all teeth were traced along the root canal for single-rooted teeth, along the palatal root canal for upper molars and the averaged course of root canals for upper bicuspids (buccal/palatal) and lower molars (mesial/distal) [28]. Apical reference points were the midpoint of the incisal edge for incisors and canines and the deepest point of the fissura for premolars and molars. Apical reference points were the most apical point of the straight part of the pulp (to exclude root curves) or the point placed at the furcation of multi-rooted teeth. The mesio-distal tip was measured as the mesial angle between the long axis of the tooth and the respective reference lines for each jaw. Regarding statistical comparisons, a smaller amount of the measured angle corresponds to a greater extent of mesial tip, whereas a greater angle indicates a higher amount of distal tip.

### 2.3. Three-Dimensional Tip and Torque Measurements Using Digital Analysis of Digitized Models

**Figure 2:**
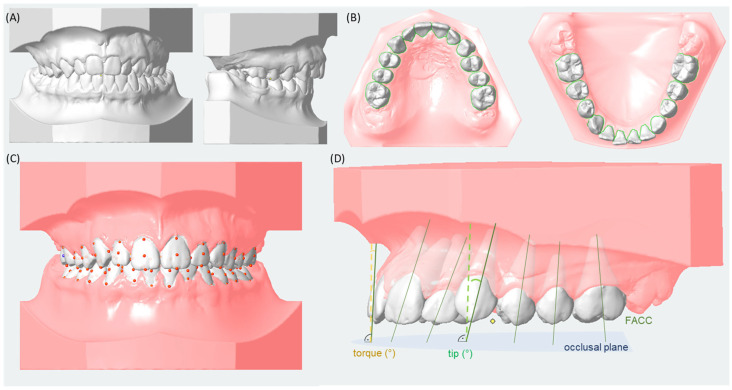
Graphical visualization of the digital three-dimensional evaluation of dental models using ‘Onyx Ceph3TM’ (Image Instruments GmbH, Chemnitz, Germany). (**A**) Alignment of models; (**B**) semi-automatically determination of the clinical crowns of each tooth; (**C**) determination of the facial axis of the clinical crown (FACC) using two reference point (incisal/occlusal and gingival) for each tooth; (**D**) measurement of tip and torque values (°) between the FACC and a perpendicular to the occlusal plane.

Plaster models (alginate impressions) of the upper and lower jaw were utilized and digitized at maximum intercuspidation using the ‘S 300 Ortho’ model scanner (Zirkonzahn Worldwide, Gais, Italy) and the ‘scan&match’ tool. The three-dimensional data (.stl data) were imported and processed in the diagnostic software ‘OnyxCeph3™’ (Image Instruments GmbH, Chemnitz, Germany; version 3.2.52). The models were aligned using the raphe palatina of the upper jaw for midline determination as well as the intercuspation of the first molars and the inclination of the jaw bases for orientation along the occlusal plane (Figure 2A).

Using the segmentation module, each tooth was marked by the examiner, and the software recognized the clinical crowns of the respective teeth semi-automatically (Figure 2B). Adjustments regarding the margins of the clinical crowns were made by the examiner. During this step, the system generates a separate coordinate system for each individual tooth and sets reference points on the clinical crown to determine the facial axis of the clinical crown: (i) the incisal/occlusal point, i.e., the midpoint of the incisal edge (incisors, canines), the cusp tip (premolars) or the facial groove between the two largest buccal cusps (molars); and (ii) the gingival point, i.e., the most gingival point of the clinical crown (Figure 2C). The occlusal plane was defined by the junction of the contact point of the lower central incisors and the mesio-buccal cusp tips of the lower first molar of the right and left side. The torque and tip values (°) were calculated as the angle between the FACC and a line perpendicular to the occlusal plane (Figure 2D). Regarding statistical comparisons of tips of teeth, a greater measured angle indicates a greater extent of mesial tip, while a smaller angle corresponds to a greater extent of distal tip. With respect to statistical comparisons of torque of teeth, a higher measured angle corresponds to a more proclined or buccal tipped position, while smaller angles indicate a more retroinclined or lingual/palatal tipped position of the tooth.

### 2.4. Statistics

The patient data and the data from the cephalometric evaluation were processed using the Excel 14.0 program (Microsoft, Redmond, WA, USA) and analyzed using the statistical program IBM SPSS Statistics Version 21 (IBM, Armonk, NY, USA). The descriptive statistics included the calculation of mean values, medians, standard deviations, minima and maxima of the collected data. The significance levels were set at *p* ≤ 0.05. Shapiro–Wilk tests were conducted for the testing of normal distribution. Non-parametric methods were utilized for variables that did not follow a normal distribution: The Kruskal–Wallis test was applied for comparisons between the groups, i.e., group 1 (class I), group 2 (class II) and group 3 (class III). If significant results were found using the Kruskal–Wallis test, statistical multiple pairwise comparisons for independent samples and Bonferroni correction were conducted. To address multiplicity, Bonferroni–Holm correction [29] was applied to tests with several significant variables, whereas Bonferroni–Hochberg correction [30] was used for tests with solely a few significant variables. Both calculations were performed using the online tool ‘MultipleTesting.com’ [31]. Post hoc power analysis was applied for comparisons between the groups (skeletal class I, II, III) using G*Power (version 3.1.9.6, Windows) [32,33]. Outcomes were calculated using the ‘F tests—ANOVA: Fixed effects, omnibus, one-way’ setting, a total sample size of n = 107 (three groups, sample size arm cI n = 36, arm cII n = 33, arm cIII n = 38) and α = 0.05. For each variable, the effect size *f* was computed based on each group’s mean value, sample size and the overall standard deviation of the complete patient cohort as reported in Supplementary Tables S3–S6. A power > 0.80 was considered as high statistical power. According to Cohen [34], an effect size *f* of *f* = 0.10 is considered a small effect, *f* = 0.25 as a medium effect, and *f* = 0.40 as a large effect. The absolute technical error of measurement (TEM) and the relative technical error of measurement (rTEM) were calculated according to Perini et al. [35] using 10 lateral cephalometric images and 10 orthopantomograms that were evaluated twice by one examiner (Supplementary Table S2). The rTEM for all measured angular variables was below 5% except for the variables ‘ANB’ (17.74%), ‘indiv. ANB’ (7.99%) and ‘PP-SN’ (11.56%), which can be explained by the small mean values of these variables. The numerical variable ‘Wits’ showed a TEM of 0.72 and a corrected rTEM of 31.0%. For classification into skeletal classes, the variable ‘Wits’ was not applied.

## 3. Results

### 3.1. Characteristics of Skeletal Class I, II and III

**Figure 3:**
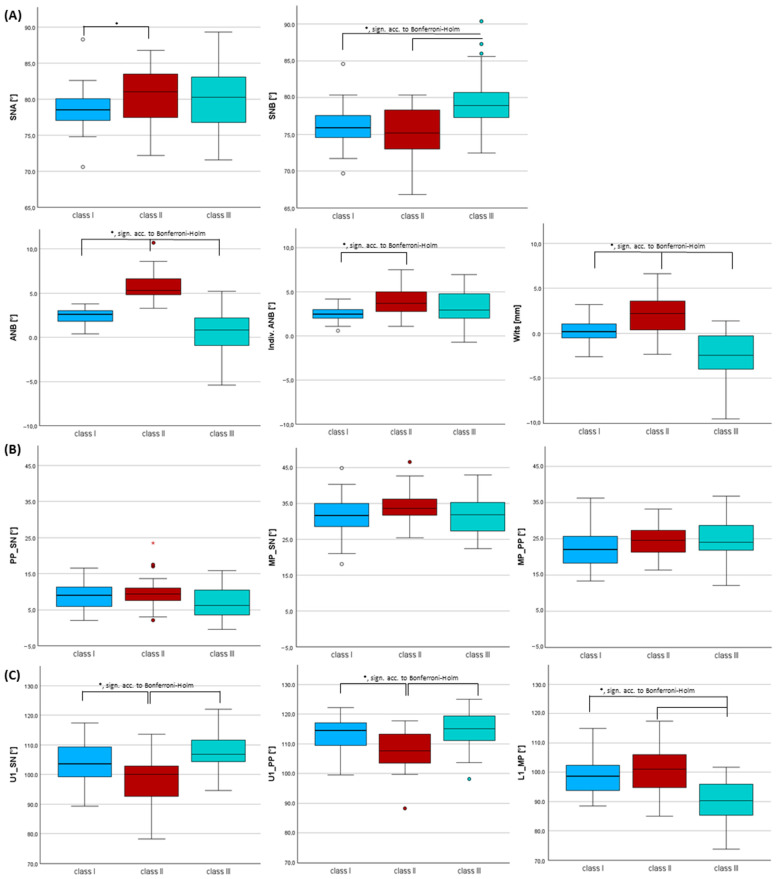
Histograms of the cephalometric evaluation: (**A**) sagittal and (**B**) vertical skeletal parameters, (**C**) inclination of incisors and a statistical comparison of the skeletal classes (I, II, III) for each tooth (UR6–UL6, LL6–LR6) and each tooth type (U1–U6, L1–L6) using Kruskal–Wallis tests (adjusted *p*-values using Bonferroni correction and multiple testing correction according to Bonferroni–Holm). * = *p* ≤ 0.005; sign. acc. to Bonferroni–Holm = *p*-value significant according to Bonferroni–Holm.

Comparing group I, II and III, significant differences (*p* ≤ 0.05) were found regarding all sagittal skeletal parameters. According to Bonferroni–Holm correction, significance was seen for all variables except the SNA angle (Figure 3, Supplementary Table S3).

Skeletal class I: Patients with skeletal class I presented an average ANB of 2.4°, which is per definition a neutral bite position (2° < ANB > 4°). Almost no difference was found between the mean ANB and the indiv. ANB (2.5°), which defined a neutral bite in accordance with Panagiotidis and Witt [26].

Skeletal class II: The average ANB angle was 5.7° in class II patients. Hence, the definition of class II regarding the ANB angle (>4°) was met. Also, the indiv. ANB angle was on average 1.7° smaller than the measured ANB angle in skeletal class II, which allowed the definition of a distal bite [26]. The highest average Wits value (1.9 mm) was also detected in class II patients, which hereby confirmed the classification of class II.

Skeletal class III: Patients with skeletal class III were characterized by a significantly greater SNB angle by 3.1° vs. skeletal class I and by 4.2° vs. class II (*p* < 0.001, sign. acc. to Bonferroni–Holm, effect size *f* = 0.471, power = 0.994). The mean ANB was 0.6°, which allowed the definition of class III (ANB < 2°). The individualized ANB angle was on average 2.5° greater than the measured ANB in skeletal class III, which allows the definition of a mesial bite [26]. Also, the mean Wits was clearly negative with an average value of −2.8 mm, validating skeletal class III.

As the rTEM was high regarding all skeletal sagittal measurements, these results must be considered with caution. However, significant differences with strong effect sizes and power > 0.80 regarding the variables used for the skeletal–jaw relationship confirmed the classification: ANB angle (*p* < 0.001, sign. acc. to Bonferroni–Holm, effect size *f* = 0.774, power 0.994), Wits (*p* < 0.001, sign. acc. to Bonferroni–Holm, effect size *f* = 0.612, power = 1.000).

### 3.2. Inclination of Incisors Using Cephalometric Measurements Within Each Skeletal Class

Patients with skeletal class II presented significantly more retroinclined upper incisors by −5.9°/−5.3° (U1-SN/U1-PP) compared to skeletal class I and by −8.8°/−6.6° compared to class III (*p* < 0.001, sign. acc. to Bonferroni–Holm, effect size *f* = 0.489/0.446, power 0.996/0.988). Patients with skeletal class III showed significantly more retroinclined lower incisors by −8.5° (L1-MP) vs. class I and by −10.9° vs. class II (*p* < 0.001, sign. acc. to Bonferroni–Holm, effect size *f* = 0.576, power 1.000) (Figure 3, Supplementary Table S3).

### 3.3. Orthopantomogram Analysis Considering Tip of the Long Axis of Teeth in Each Skeletal Class

**Figure 4:**
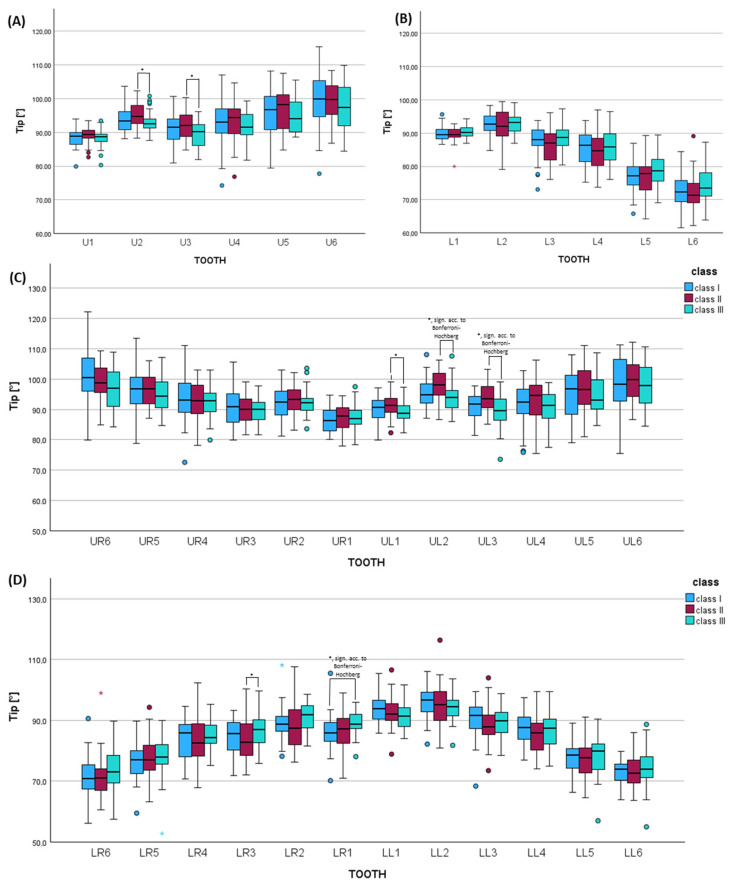
Histograms of the orthopantomogram evaluation tip of the long axis comparing the skeletal classes (I, II, III) for (**A**) each tooth type of the upper jaw (U1–U6) and (**B**) lower jaw (L1–L6) and each tooth of the (**C**) upper jaw (UR6–UL6) and (**D**) lower jaw (LL6–LR6) using Kruskal–Wallis tests (adjusted *p*-values using Bonferroni correction and multiple testing correction according to Bonferroni–Hochberg). * = *p* ≤ 0.005. sign. acc. to Bonferroni–Holm = *p*-value significant according to Bonferroni–Hochberg.

In the upper jaw, skeletal class II was characterized by greater distal tipping of the lateral incisor by 2° (U2: *p* = 0.021, not significant acc. to Bonferroni–Hochberg, effect size *f* = 0.315, power = 0.828), which was statistically significant acc. to Bonferroni–Hochberg on the left side (UL2: *p* = 0.007, sign. acc. to Bonferroni–Hochberg, effect size *f* = 0.288, power = 0.752) (Figure 4, Supplementary Table S4). Also, the upper canine was tipped more distally in class II by 2.7° compared to class III (U3: *p* = 0.050, not sign. acc. to Bonferroni–Hochberg, effect size *f* = 0.555, power = 1.000), which was statistically significant acc. to Bonferroni–Hochberg on the left side (UL3: *p* = 0.021, sign. acc. to Bonferroni–Hochberg, effect size *f* = 0.345, power = 0.893).

In the lower jaw, patients with skeletal class III showed significantly greater distal tipping of the lower right central incisor by 3.1° vs. class I (LR1: *p* = 0.007, sign. acc. to Bonferroni–Hochberg, effect size *f* = 0.269, power = 0.691) (Figure 4, Supplementary Table S4).

### 3.4. Digital Dental Cast Analysis Considering Tip of the Clinical Crown for Each Skeletal Class

**Figure 5:**
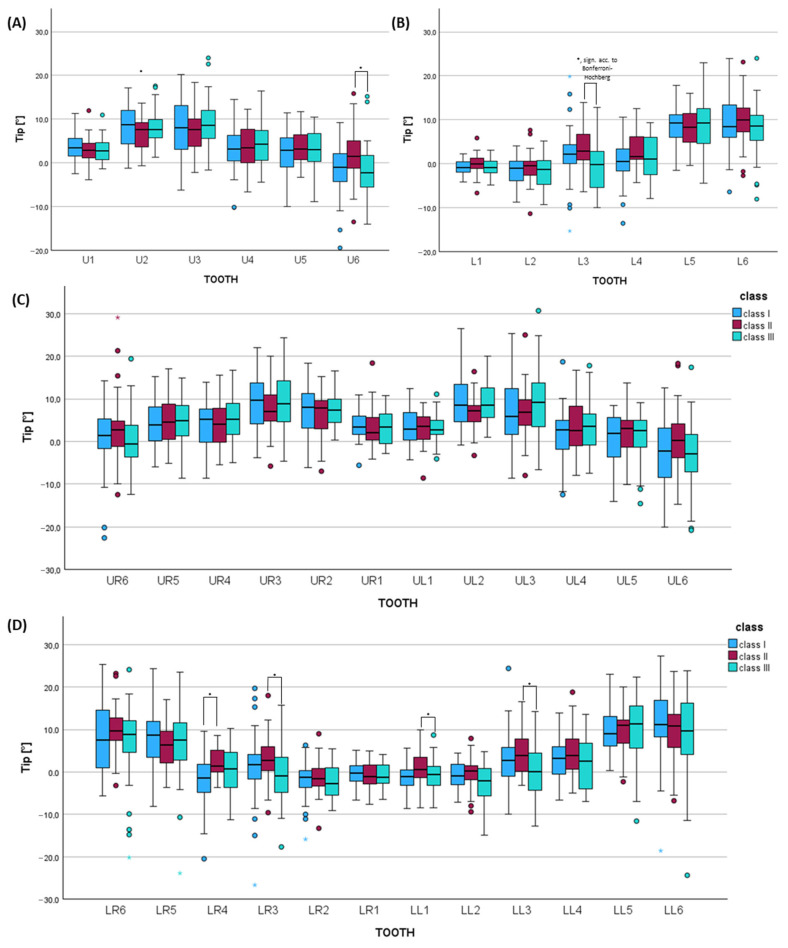
Histograms of the dental cast analysis regarding tip of the clinical crown of (**A**) each tooth type of the upper jaw (U1–U6) and (**B**) lower jaw (L1–L6) and each tooth of the (**C**) upper jaw (UR6–UL6) and (**D**) lower jaw (LL6–LR6) comparing the skeletal classes (I, II, III) using the Kruskal–Wallis test (adjusted *p*-values using Bonferroni correction and multiple testing correction according to Bonferroni–Hochberg). * = *p* ≤ 0.005. sign. acc. to Bonferroni–Holm = *p*-value significant according to Bonferroni–Hochberg.

Patients with skeletal class II presented greater mesial tipping of the upper first molar by 3.8° compared to class III (*p* = 0.020, not sign. acc. to Bonferroni–Hochberg, effect size *f* = 0.284, power = 0.739) (Figure 5, Supplementary Table S5). Class II patients were characterized by significantly greater mesial tipping of the lower canine by 3.5° I (*p* = 0.009, sign. acc. to Bonferroni–Hochberg, effect size *f* = 0.242, power = 0.591) compared to class III (Figure 5, Supplementary Table S5).

### 3.5. Digital Dental Cast Analysis Considering Torque of the Clinical Crown for Each Skeletal Class

**Figure 6:**
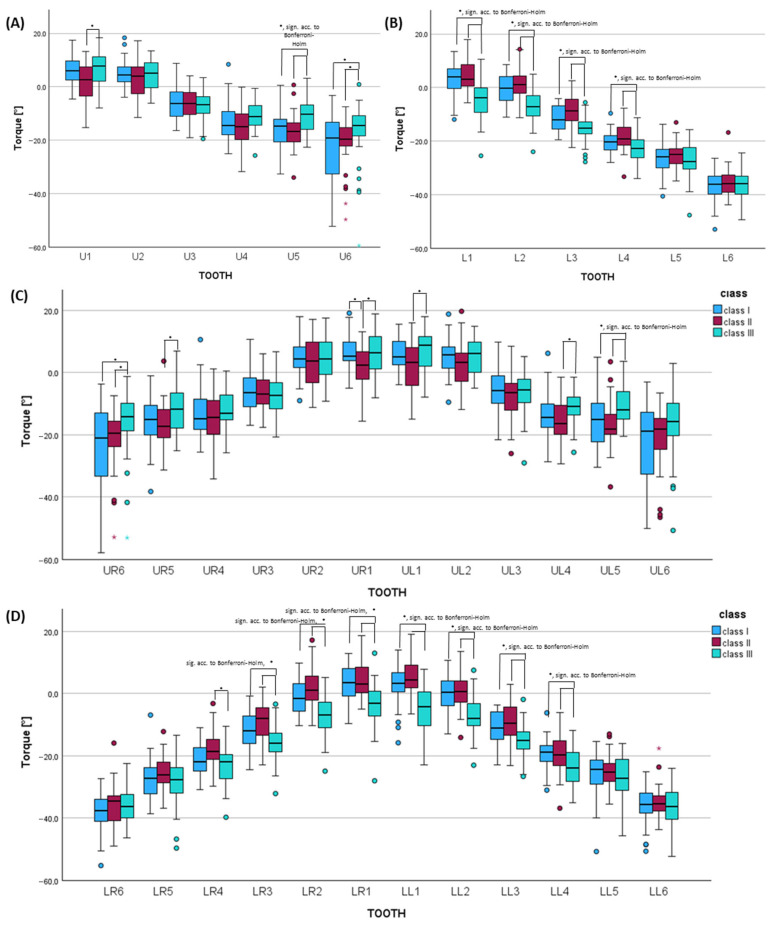
Histograms of the dental cast analysis regarding torque of the clinical crown of (**A**) each tooth type of the upper jaw (U1–U6) and (**B**) lower jaw (L1–L6) and each tooth of the (**C**) upper jaw (UR6–UL6) and (**D**) lower jaw (LL6–LR6) comparing the skeletal classes (I, II, III) using the Kruskal–Wallis test (adjusted *p*-values using Bonferroni correction and multiple testing correction according to Bonferroni–Holm). * = *p* ≤ 0.005. sign. acc. to Bonferroni–Holm = *p*-value significant according to Bonferroni–Holm.

Considering the upper jaw, patients with skeletal class II showed greater retroinclination of the upper central incisor by −4.2° (*p* = 0.015, not sign. acc. to Bonferroni–Holm, effect size *f* = 0.286, power 0.745) compared to class III (Figure 6, Supplementary Table S6). The first premolar was tipped buccally by 4.0° compared to class II (*p* = 0.028, not sign. acc. to Bonferroni–Holm, effect size *f* = 0.251, power 0.623). Also, the second premolar of class III patients showed significantly greater buccal tipping by 5.9° vs. class II and by 4.6° vs. class I (*p* = 0.001, significant acc. to Bonferroni–Holm, effect size *f* = 0.355, power 0.910). Class III patients also showed greatest buccal tipping of the upper first molars by 4.6° compared to class I and by 3.6° vs. class II (*p* = 0.015, not sign. acc. to Bonferroni–Holm, effect size *f* = 0.175, power 0.339).

In the lower jaw, class III patients were characterized by significantly greater retroinclination of the central and lateral incisors by −8.9°/8.5° compared to class II and by −7.0°/−6.0° compared to class I (L1: *p* < 0.001, significant acc. to Bonferroni–Holm, effect size *f* = 0.522, power 0.999; L2: *p* < 0.001, significant acc. to Bonferroni–Holm, effect size *f* = 0.527, power 0.999). Class III was also characterized by significantly larger lingual tipping of the lower canine by −6.4° vs. class II and −3.8° vs. class I (*p* < 0.001, significant acc. to Bonferroni–Holm, effect size *f* = 0.446, power 0.988). Further, the first premolars of class III patients were tipped significantly more lingually, i.e., by −4.0° compared to class II and −2.6° compared to class I (*p* < 0.001, significant acc. to Bonferroni–Holm, effect size *f* = 0.326, power 0.846).

## 4. Discussion

The overarching goal of this study was to investigate the tip and torque of teeth before orthodontic treatment with skeletal class I, II and III using cephalometric (A), orthopantomogram (B) and digital dental cast analysis (C). Significant differences between the skeletal classes were found:

The following demonstrates that skeletal class II was characterized by significantly (*p* ≤ 0.05, sig. acc. to Bonferroni–Holm/Hochberg)

(1) More retroinclined upper central incisors by ~7–9° vs. class III and by~−5–6° vs. class I (A);
(2) More proclined lower incisors by ~11° compared to class III (A), which was also seen for the central and lateral incisors by 8–9° vs. class III (C);
(3) Greater mesial tipping of lower canines by 3.5° vs. class III (C);
(4) Greater palatal tipping of the upper second premolars by ~−6° vs. class III (C);

Skeletal class III was characterized by significantly (*p* ≤ 0.05, sig. acc. to Bonferroni–Holm/Hochberg);

(1) Greater proclination of upper central incisors by ~8° vs. class II (A);
(2) More retroinclined incisors by ~−11° vs. class II and −8–9° vs. class I (A), which was also seen for the central and lateral incisors by −8–9° vs. class III and −6–7° vs. class I (C);
(3) Greater distal tipping of lower canines by 3.5° vs. class II (C);
(4) Greater buccal tipping of the upper second premolar by ~6° vs. class II (C);
(5) Higher lingual tipping of canines and the first premolar in the lower jaw by −6.4° and −4.0° vs. class II (C).

Accordingly, differences regarding the incisor inclination were described: Hernández-Sayago et al. [13] performed a retrospective cephalometric study and detected that class II patients were characterized by proclination of the lower incisors and class III patients by retroinclination of the lower incisors. A retrospective study by Santana et al. [14], in which torque of the upper molars were investigated using digitized models, showed that palatal tipping increased progressively by ~6° between ages 9 and 14 years in patients with class II [14]. This implicates that dental compensation mechanisms in terms of palatal tipping increase during growth. Busato et al. [36] also detected greater mesial tipping of the lower canines in skeletal class II. Similarly, Su et al. [37] detected differences regarding the upper first molar with greater distal tipping in the upper jaw and greater mesial tipping in the lower jaw in class II and, vice versa, in class III.

Regarding the tip measurements using two-dimensional and three-dimensional measurements, the investigation methods presented similar results regarding class-specific differences for most teeth; however, some differences were found (Supplementary Table S5). On the one hand, this can be explained by the analyzed tooth axis: the long axis of the teeth was used for orthopantomogram measurements and the clinical crown axis for dental cast analysis. Also, different reference planes are used. While orthopantomogram analysis used anatomical points (orbitae, foramen mental) for the reference line, the dental cast analysis used the occlusal plane as a reference plane. As the occlusal plane might be affected by the skeletal class [38], an impact of the occlusal plane on the tip measurement in different classes must be expected. Differences can also be explained by the limitations of orthopantomogram evaluations for tip measurements due to projection distortion and variable magnifications. A recent study by Bouwens et al. [39] found significant differences between the tips of teeth measured using orthopantomograms and cone-beam computed tomography. The authors [39] suggest including examinations of the tip in the evaluation of mesio-distal angulation seen in panoramic images. Therefore, the detected significant differences found using orthopantomograms should be interpreted with caution as the three-dimensional measurements (digital dental cast analysis) do not support all findings. On the other hand, some results of both measurements were strengthened as they were detected using both methods. For example, considering the comparisons of tip differences between the skeletal classes (Supplementary Table S5), both methods detected distal tipping of the upper incisors, canines and first premolars as well as greater mesial tipping of the lower incisors, canines, first premolars and molars in skeletal class II compared to skeletal class III. Similarly, comparisons of both methods revealed similar overall results regarding the upper and lower incisors, upper and lower canines as well as lower molars between skeletal class I and II/III, indicating good reliability of the orthopantomogram measurements for those teeth. However, differences were seen regarding the upper molars and sometimes referring to the first and/or second premolar. For clinical practice, orthopantomogram analyses of tips can be a useful tool regarding incisors and canines (and lower molars). However, for premolars and upper molars dental cast interpretation is suggested.

Limitations: The retrospective design of study is a weakening aspect. Yet, results of retrospective studies can pave the way for future prospective studies [40,41]. Also, as no treatment results were studied, no benefit would result in choosing patients with skeletal class I, II and III prospectively. Another potential bias is that torque measurements (dental casts) were referenced to the occlusal plane. It was shown that the angle between the occlusal plane and the AB plane allowed a differentiation between class I and III [38]. The angles between the occlusal plane and the AB line as well as to the FH plane were significantly greater in class III compared to class I, i.e., a steeper occlusal plane in class III [38]. Another study showed that the angles between the anterior and posterior occlusal plane and the SN line were greater in children with skeletal class II vs. class III in the mixed dentition [42]. Those differences in the occlusal plane orientation between the skeletal classes can potentially impact the results detected in this study. Another point of criticism is the very high rTEM regarding the variables ‘ANB’ (17.74%), ‘indiv. ANB’ (7.99%), ‘PP-SN’ (11.56%) and ‘Wits’ (31.0%). All reference points were set by one examiner. Hence, due to the high rTEM of the aforementioned measurements, the intra-rater reliability for the parameters ‘ANB’, ‘indiv. ANB’, ‘PP-SN’ and ‘Wits’ was poor. This can be explained by relatively small values, which leads to higher rTEM percentages [40]. Reference points are reproducible to varying degrees: (i) Sella and Nasion are usually easy to determine [43,44]; (ii) point B and A show great variation as their position is influenced by the periodontium of the central incisors [43,44]; (iii) reference points on the median sagittal plane are easier to determine than bilateral points as—even with the patient’s head in the optimal position in the cephalostat—double contours arise due to the different distances of bilateral points from the image plane [45]; (iv) reproducibility of the occlusal plane and inclination of incisors is most difficult [43,44,45]. Also, a recent study comparing analog to digital cephalometric evaluation showed that the reproducibility of longitudinal measurements of the skeletal–jaw relationship were poor regarding both methods [46]. Overall, a high rTEM regarding the skeletal sagittal parameters ‘ANB’, ‘indiv. ANB’ and ‘Wits’ was expected. As the variables ‘ANB’, ‘indiv. ANB’ and ‘Wits’ were used for verification of skeletal classes, the classification into skeletal class I, II and III was performed twice and independently of each other: (i) for orthodontic treatment planning as all patients were treated in the Department of Orthodontics and Dentofacial Orthopedics, University Hospital, LMU Munich; and (ii) in this study for verification of the three groups (skeletal class I; II; III). Another limitation of the study is that adolescent patients with functional pretreatment/phase I therapy were not excluded. It is known that pretreatment for growth modification not only shows skeletal but also dental side effects, which can alter tooth axes. For example, functional pretreatment with Sander-II appliance effectively reduces the ANB angle by ventral positioning of the mandible and growth restriction of the maxilla. However, its dental side effect is the retrusion of upper incisors [6]. Phase I therapy using a face mask for maxillary protraction can lead to mesial migration of upper lateral teeth and protrusion of the upper incisors [7]. Hence, some of the detected compensation mechanisms are due to functional pretreatment. While the long-term effects of phase I or functional pre-treatment are discussed in the literature [47], pretreatment with functional appliances prior to orthodontic treatment is a common treatment strategy among orthodontists. For example, the Twin Block appliance is the most popular appliance in the UK [48], and a recent survey revealed that 99% of the participating orthodontists in the UK use the Twin Block appliance [49]. Patients with pretreatment were included to mimic the patient collective of patients with skeletal dysgnathia receiving neither surgical nor camouflage extraction therapy. The patients included either showed naturally occurring or treatment-associated dental compensation. Either way, the treatment goal should be to maintain these dental compensations to some extent, as decompensation is not desired in those non-surgical cases as it could alter the treatment results.

One strength of the study is its methodology, as several methods for the investigation of the tip and torque of teeth were applied. In everyday clinical practice, it is common to determine the inclination of the incisors using cephalometrics, in which the longitudinal axis of the tooth is determined based on the pulp line, the incisal tip and the apex. As discussed in a previous study by Seidel et al. [40], repeatability, reliability and reproducibility of cephalometric, digital measurements were shown by several studies [46,50,51,52]. Also, validity of three-dimensional dental cast analysis using scanned plaster models was found to be comparable with examinations of digital models or plaster models [53,54]. In daily practice, orthopantomograms are part of standard orthodontic diagnostics prior to and during treatment with fixed appliances [39]. Orthodontists use these panoramic images for bracket placement and for refinement of treatment results prior to debonding of the appliance. As differences between two-dimensional and three-dimensional X-ray analysis were shown regarding tip of teeth, and usage of both panoramic and clinical examination were suggested [39], this study adds the evaluation of tip using three-dimensional dental casts. Also, the torque of incisors was evaluated using cephalometrics and dental cast analysis. By using two methods, results are either strengthened when found with two methods or weakened when solely detected by one method. As dental cast analysis only determines the inclination of the clinical crown and cephalometrics analyze the long axis of the incisors, the measurement cannot be compared directly [8]. Yet, Andrews [55] found that the inclination measured using cephalometrics differs by +18° for the upper incisor and +16° for the lower central incisor, compared to the FACC in relation to the perpendicular occlusal plane, and proposed a subtraction method for comparisons. Variations between 7 and 24° depending on the shape of the tooth can be found between the clinical crown and the longitudinal axis of the tooth [56,57]. Knösel et al. [56] also showed a correlation between both methods but concluded that the dental cast analysis was more precise and determined values were clinically easier to apply in the straight-wire appliance. In this study, U1-SN was 104.5° in class I, 98.6° in class II and 107.4° for class III, and L1-MP was 98.7° in class I, 101.4° in class II and 90.5° in class III. The torque of the clinical crown of U1 was 6.2° for class I, 2.4° for class II and 6.6° for class III, and L1 2.9° for class I, 4.8° for class II and −4.1° for class III. While the number of differences were greater using cephalometric analyses, differences between the skeletal classes were confirmed using both methods. In contrast, Andrews [8] only performed dental cast analysis, and there are several methodical factors that might contribute to differences found: (i) cast analyses only take into account the crown axis, not the entire longitudinal axis determined using cephalometrics [9]. and varying degrees of tip between the root and crown axes can lead to false interpretations of the inclination [56]; (ii) measurements were not repeated. Hence, the relative technical error of measurements cannot be detected; (iii) selection criteria of the patient collective was not sufficiently detailed or described; (iv) neither gender, ethnic group, age-related differences, individual tooth size, nor asymmetry of the respective jaws were taken into account in the measurements [58]. While there are novel three-dimensional studies using cone-beam computed tomography scans for investigation of tooth axes [12,15,59], none of them considered the tip and torque of all teeth of the upper and lower jaw in different skeletal classes: (i) Wang et al. [12] considered tip and torque of all teeth; however, solely in ‘normal occlusion’. (ii) In another study by Wang et al. [15], differences between skeletal classes were considered; however, only regarding the upper and lower incisors. (iii) Coşkun and Kaya [59] investigated the torque of teeth and compared it between the skeletal classes. However, tip was not evaluated. In contrast to cone-beam computed tomography scans, lateral cephalometric radiographs, orthopantomograms and dental casts are part of the standard orthodontic diagnostic documents. In daily practice, orthodontists will not use cone-beam computed tomography scans for treatment decisions regarding tooth axes, e.g., bracket placement and decision of debonding. Therefore, the results of our studies using standard orthodontic diagnostic documents are clinically more relevant than investigations using cone-beam computed tomography.

Clinical implications: Compensation mechanisms in skeletal class II and class III were particularly evident regarding incisor inclination/torque. This is clinically highly relevant as a change in upper incisor inclination was shown to alter molar relationship [60]. It was shown that orthodontic treatment with buccal appliances (self-ligating brackets) leads to an average proclination of the incisors by 5° [40]. In class III patients, lower incisors are significantly more retroinclined compared to class I and II. Orthodontic treatment with self-ligating brackets would decompensate the axes of lower incisors and potentially result in anterior cross bite. Treatment planning in class III can be adapted by increased anchorage and, e.g., by not including lower incisors during leveling and aligning or by choosing standard edgewise brackets for the lower front teeth. Class II patients show more proclined lower incisors compared to class III: In the treatment of class II malocclusion, a further enhancement of the protruded lower incisors can lead to an early contact in the front. This can impede anterior placement of the mandible and correction of distal occlusion. Therefore, treatment of skeletal class II prior to orthodontic treatment, e.g., by functional appliances like the Sander-II appliance [6], are suggested. Also, this study found that males show greater lower incisor inclination. As it was shown that orthodontic treatment increases proclination in males more than in females [40], clinicians must consider biomechanical adaptations of orthodontic treatment in males, e.g., increased anchorage in the lower jaw or methods to generate space (approximal enamel reduction). In this study, class II patients showed significantly greater mesial tipping of lower canines and a tendency towards greater mesial tipping of incisors and lateral teeth. Hence, class II mechanics used during orthodontic treatment leading to mesially directed forces in the lower jaw (e.g., class II elastics) should be applied carefully and only for a short period of time to avoid anchorage loss and increase in proclination and mesial tipping of lower canines and lateral teeth. Similarly, as class III patients have greater mesial tipping of upper lateral teeth and canines, class III mechanics with mesially directed forces in the upper jaw (class III elastics, Delaire mask) must be applied with caution. Also, mesial tipping of upper canines can be accompanied by anchorage loss and mesial migration of lateral teeth. Therefore, in class II patients, increased anchorage must be applied in the upper jaw—not only regarding the first molars, but also for the canines, e.g., by interlocking using steel ligatures. This study also found that class II patients have greater palatal tipping of the upper premolars and the first molar compared to class III and, vice versa, class III patients have greater buccal tipping of those teeth. Also, class III patients show greater lingual tipping of lower canines and premolars in the lower jaw. Depending on the skeletal transversal parameters, transversal anchorage systems or adaptations of applied bracket prescription might be necessary. If no buccal tipping of the upper first molar in class II is desired, increased anchorage (transpalatal arch, quadhelix) should be applied.

## 5. Conclusions

The comparison of tip and torque of the long axis of the teeth and the clinical crown between patients with skeletal class I, II and III prior to orthodontic treatment revealed the following: Compensation mechanisms in skeletal class II regarding overjet, distal occlusion and skeletal sagittal discrepancy were seen by significantly more retroinclined upper central incisors by approximately 7° compared to class III and 5° compared to class I as well as significantly more proclined lower incisors by approx. 9° compared to class III. Also, greater mesial tipping of lower canines by 3.5° compared to class III was found. Also, palatal tipping of the upper first premolar by ~6° compared to class III was found.

Compensation mechanisms in skeletal class III considering overjet, mesial occlusion and skeletal sagittal discrepancy were seen by significantly greater proclination of upper central incisors by approx. 7° compared to class II and significantly more retroinclined central and lateral lower incisors by approx. 9° vs. class II and 7° vs. class I. Also, greater distal tipping of lower canines by 3.5° was seen compared to class II. Greater buccal tipping of the upper second premolar by ~6° compared to class II was seen. Also, higher lingual tipping of lower canines by 6–7° compared to class II and 4° compared to class I and greater lingual tipping of the lower first premolar by 4° vs. class II and 3° vs. class I was detected.

Overall, the evaluation of tip and torque regarding different measurement methods revealed differences regarding tip measurements using orthopantomogram vs. Three-dimensional dental cast analyses. Results detected using solely orthopantomogram analyses should be therefore interpreted with caution. Also, orthodontists should consider whether usage of orthopantomograms for evaluation of tip of teeth for bracket placement and before debonding is ideal. A limitation of the study is that the rTEM of skeletal sagittal parameters was high; therefore, differences between the skeletal classes should be validated in future studies. In clinical practice, orthodontists must consider the compensation mechanisms in skeletal class II and class III patients regarding tip and torque of teeth in biomechanical treatment planning.

## Data Availability

Data is unavailable due to privacy and ethical restrictions.

## Supplementary Materials

The following supporting information can be downloaded at https://www.mdpi.com/article/10.3390/jcm14217738/s1: This file contains Supplementary Table S1: Cephalometric measurements regarding (a) reference points, (b) reference lines and (c) angular measurements. Supplementary Table S2: Statistical evaluation of the absolute technical error of measurement (TEM) and the relative technical error of measurement (rTEM): variables of (a) the cephalometric analysis, (b) orthopantomogram analysis. Supplementary Table S3: Results of the cephalometric evaluation (sagittal and vertical skeletal parameters, inclination of incisors) regarding the total study population and a comparison of the skeletal classes (I, II, III) for each tooth (UR6–UL6, LL6–LR6) and each tooth type (U1–U6, L1–L6). Supplementary Table S4: Results of the orthopantomogram evaluation tip of the long axis regarding the total study population and a comparison of the skeletal classes (I, II, III) for each tooth (UR6–UL6, LL6–LR6) and each tooth type (U1–U6, L1–L6). Supplementary Table S5: Results of the dental cast analysis considering tip of the clinical crown regarding all teeth of the total study population and a statistical comparison (Kruskal–Wallis test) between the different skeletal classes (I, II, III). Supplementary Table S6: Results of the dental cast analysis considering torque of the clinical crown regarding all teeth of the total study population and a statistical comparison (Kruskal–Wallis test) between the different skeletal classes (I, II, III). Supplementary Table S7: Interpretation of differences regarding measured tip of teeth considering the (a) orthopantomogram evaluation of the long axis and (b) dental cast analysis of the clinical crown between the different skeletal classes (I, II, III).
