# Supplementary material for: A Retrospective, Digital Evaluation of Tip and Torque of Teeth in Patients with Skeletal Class I, II and III Using Lateral Cephalograms, Orthopantomograms and Digitized Models
Source: J Clin Med. 2025 Oct 31;14(21):7738. doi: 10.3390/jcm14217738 (PMC12610279; doi:10.3390/jcm14217738)
Supplement: Supplementary file 1 [file jcm-14-07738-s001.zip › jcm-3911905-supplementary.pdf]

Supplementary Table S1: Cephalometric measurements regarding (a) reference points, (b) reference lines and (c) angular measurements.

Variable	Definition
**(a) Reference points**	
Nasion (N)	The most anterior point of the frontonasal suture
Sella (S)	The midpoint of sella turcica
Menton (Me)	The most inferior point of the mandibular symphysis
Gonion (Go)	The most posterior. inferior point of the mandibular angle
A point (A)	The deepest point of the curvature of the maxillary alveolar process
B point (B)	The deepest point of the curvature of the mandibulary alveolar process
Anterior nasal spine (ANS)	The most anterior point of the nasal spine of the palate
Posterior nasal spine (PNS)	The most posterior point of the nasal spine of the palate
Incision superior (is)	incisal edge of the most anterior maxillary central incisor
Apicale superior (as)	apex of the most anterior maxillary central incisor
Incision inferior (ii)	incisal edge of the most anterior mandibular central incisor
Apicale inferior (ia)	apex of the most anterior mandibulary central incisor
**(b) Reference lines**	
sella-nasion plane (SN)	connecting line between point S and point N
palatal plane (PP)	connecting line between ANS and PNS
mandibular plane (MP)	connecting line between Me and Go
Upper Incisor (U1)	connecting line between is and as; long axis of U1
Lower Incisor (L1)	connecting line between ii and ia; long axis of L1
**(c) Cephalometric measurements**	
SNA (°)	angle between S-N and point A; antero-posterior position of the maxilla to the cranial base
SNB (°)	angle between S-N and point B; antero-posterior position of the mandibula to cranial base
ANB (°)	angle between point A. N and B; sagittal position between maxilla and mandibula
PP-SN (°)	angle between the planes PP and SN; vertical position of the maxilla to the cranial base
MP-SN (°)	angle between the planes MP and SN; vertical position of the mandibula to the cranial base
MP-PP (°)	angle between the planes MP and PP; interbase angle between maxilla and mandibula
U1-SN (°)	angle between the long axis of U1 and SN; inclination of U1 to the cranial base
U1-PP (°)	angle between the long axis of U1 and PP; inclination of U1 to the palatal plane
L1-MP (°)	angle between the L1 and MP; inclination of L1 to the mandibulary base

Supplementary Table S2: Statistical evaluation of the absolute technical error of measurement (TEM) and the relative technical error of measurement (rTEM): variables of a) the cephalometric analysis and b) orthopantomogram analysis.

Cephalometric analysis			Orthopantomogram analysis					
**Variable**	**TEM**	**rTEM**	**Variable**	**TEM**	**rTEM**	**Variable**	**TEM**	**rTEM**
SNA (°)	0.87	1.08	UR1	1.16	1.33	LL1	1.40	1.54
SNB (°)	0.52	0.67	UR2	1.18	1.28	LL2	1.46	1.59
ANB (°)	0.61	17.74	UR3	1.14	1.28	LL3	1.78	2.00
Indiv. ANB (°)	0.28	7.99	UR4	2.15	2.37	LL4	2.41	2.74
MP-SN (°)	0.55	1.70	UR5	3.15	3.35	LL5	1.31	1.64
PP-SN (°)	0.97	11.56	UR6	2.41	2.49	LL6	2.86	3.87
MP-PP (°)	1.05	4.38	UL1	1.26	1.38	LR1	1.56	1.78
U1-SN (°)	1.30	1.24	UL2	1.31	1.37	LR2	1.12	1.27
U1-PP (°)	1.40	1.23	UL3	1.74	1.89	LR3	1.21	1.42
L1-MP (°)	1.37	1.39	UL4	4.12	4.64	LR4	1.02	1.18
Wits	0.72	31.00	UL5	1.36	1.46	LR5	1.75	2.20
			UL6	3.20	3.26	LR6	1.35	1.84

Supplementary Table S3: Results of the cephalometric evaluation (sagittal and vertical skeletal parameters, inclination of incisors) regarding the total study population and a comparison of the skeletal classes (I, II, III) for each tooth (UR6-UL6, LL6-LR6) and each tooth type (U1-U6, L1-L6).

Variable	Study population		Skeletal classes /groups									Statistical tests						
	Total (n=107)		Class I (n=36)		Class II (n=33)		Class III (n=38)		Differences (MV)			Kruskal-Wallis test (total n = 107)				Bonferroni-Holm-correction $p_{B\text{-}HOLM} \leq 0.000991112$	Effect size *f*	Power
	MV	SD	MV	SD	MV	SD	MV	SD	cII-III	cII-I	cIII-I	*p*-value	cII-III*	cII-I*	cIII-I*			
SNA (°)	79.7	4.1	78.5	2.9	80.8	4.2	79.9	4.7	0.9	2.3	1.4	**0.048451893**	1.000	**0.045**	0.374	n. s.	0.228	0.536
SNB (°)	77.0	3.8	76.2	2.6	75.1	3.8	79.3	3.8	-4.2	-1.1	3.1	**0.000004923210623**	**<0.001**	1.000	**<0.001**	significant	0.471	*0.994*
ANB (°)	2.8	2.7	2.4	0.9	5.7	1.4	0.6	2.4	5.1	3.3	-1.8	**0.0000000000000003997**	**<0.001**	**<0.001**	0.035	significant	0.774	*1.000*
Indiv. ANB (°)	3.2	1.6	2.5	0.8	4.0	1.6	3.1	2.0	0.9	1.5	0.6	**0.000991112**	0.155	**0.001**	0.187	significant	0.378	*0.942*
Wits (mm)	-0.3	3.2	0.3	1.4	1.9	2.3	-2.8	3.4	4.7	1.6	-3.1	**0.0000000000080044**	**<0.001**	0.022	**<0.001**	significant	0.612	*1.000*
PP-SN (°)	8.7	4.1	9.0	3.7	9.6	4.4	7.6	4.1	2	0.6	-1.4	0.128766111	0.197	1.000	0.317	n. s.	0.205	0.451
MP-SN (°)	32.5	5.5	31.3	5.6	34.7	4.9	31.9	5.5	2.8	3.4	0.6	0.071939592	0.163	0.113	1.000	n. s.	0.264	0.670
MP-PP (°)	23.8	4.9	22.3	5.0	25.0	4.6	24.3	4.8	0.7	2.7	2	0.051202186	1.000	0.071	0.169	n. s.	0.232	0.551
U1-SN (°)	103.8	7.4	104.5	6.2	98.6	7.5	107.4	6.0	-8.8	-5.9	2.9	**0.000005716402202**	**<0.001**	**0.006**	0.226	significant	0.489	*0.996*
U1-PP (°)	112.5	6.3	113.6	5.4	108.3	5.5	114.9	6.2	-6.6	-5.3	1.3	**0.000053149734077**	**<0.001**	**0.002**	1.000	significant	0.446	*0.988*
L1-MP (°)	96.6	8.1	98.7	5.9	101.4	7.4	90.5	6.8	10.9	2.7	-8.2	**0.000000071879**	**<0.001**	1.000	**<0.001**	significant	0.576	*1.000*

MV = mean value; SD = standard deviation; bold letters = significant p-values (p<0.05); n. s. = not significant; italic letters = power (>0.80)

*p-values of pairwise comparisons after Bonferroni-correction as implemented in SPSS Version 29

Supplementary Table S4: Results of the orthopantomogram evaluation tip of the long axis regarding the total study population and a comparison of the skeletal classes (I, II, III) for each tooth (UR6-UL6, LL6-LR6) and each tooth type (U1-U6, L1-L6).

Tooth	Study population		Skeletal classes /groups									Statistical tests						
	Total (n=107)		Class I (n=36)		Class II (n=33)		Class III (n=38)		Differences (MV)			Kruskal-Wallis test (Total n = 107)				Bonferroni-Hochberg-correction	Effect size *f*	Power
	MV	SD	MV	SD	MV	SD	MV	SD	cII-III	cII-I	cIII-I	*p*-values	cII-III*	cII-I*	cIII-I*	$p_{BH} \leq 0.007066933$		
UR1	87.0	4.2	86.3	4.0	87.1	4.5	87.5	4.2	-0.4	0.8	1.2	0.424149571	1.000	1.000	0.609	n. s.	0.120	0.179
UR2	92.4	4.6	91.9	5.3	93.0	4.6	92.3	4.0	0.7	1.1	0.4	0.642227348	1.000	1.000	1.000	n. s.	0.097	0.130
UR3	90.2	4.8	90.5	5.7	90.6	4.7	89.5	3.8	1.1	0.1	-1	0.643586635	1.000	1.000	1.000	n. s.	0.105	0.145
UR4	93.0	6.1	93.5	7.3	93.3	5.6	92.4	5.0	0.9	-0.2	-1.1	0.728004258	1.000	1.000	1.000	n. s.	0.080	0.103
UR5	95.9	6.3	95.9	7.3	96.9	5.7	95.1	5.7	1.8	1	-0.8	0.521392762	0.888	1.000	1.000	n. s.	0.116	0.169
UR6	98.8	7.3	100.6	8.5	99.1	5.8	97.0	6.9	2.1	-1.5	-3.6	0.078921798	0.630	1.000	0.075	n. s.	0.206	0.453
UL1	90.0	3.7	90.1	3.8	91.0	3.9	89.0	3.1	2	0.9	-1.1	**0.016069657**	**0.016**	1.000	0.162	n. s.	0.221	0.509
UL2	95.8	4.9	95.2	4.6	97.9	5.1	94.6	4.6	3.3	2.7	-0.6	**0.006726116**	**0.005**	0.132	0.789	significant	0.288	0.752
UL3	91.4	5.1	90.7	4.5	94.1	4.7	90.0	5.1	4.1	3.4	-0.7	**0.004991626**	**0.005**	0.058	1.000	significant	0.345	*0.893*
UL4	91.6	6.1	91.5	6.6	93.3	5.9	90.3	5.6	3	1.8	-1.2	0.107799317	0.110	1.000	0.623	n. s.	0.200	0.431
UL5	95.4	6.6	94.6	7.5	96.9	6.2	95.0	5.9	1.9	2.3	0.4	0.334811889	0.464	0.822	1.000	n. s.	0.149	0.254
UL6	98.5	7.5	98.2	8.4	99.7	6.7	97.8	7.3	1.9	1.5	-0.4	0.422376642	0.571	1.000	1.000	n. s.	0.107	0.151
LL1	92.7	4.6	93.7	4.6	93.0	5.3	91.5	3.9	1.5	-0.7	-2.2	0.102348351	0.649	1.000	0.102	n. s.	0.203	0.443
LL2	95.5	5.7	96.3	5.3	95.3	7.2	94.7	4.6	0.6	-1	-1.6	0.245358949	1.000	0.498	0.396	n. s.	0.118	0.173
LL3	89.6	5.8	90.1	6.0	88.4	6.3	90.1	5.1	-1.7	-1.7	0	0.175956641	0.517	0.216	1.000	n. s.	0.135	0.217
LL4	86.7	5.8	87.1	5.2	85.6	6.5	87.2	5.9	-1.6	-1.5	0.1	0.463396086	0.910	0.773	1.000	n. s.	0.124	0.187
LL5	77.9	6.2	77.9	5.1	76.9	6.7	78.9	6.6	-2	-1	1	0.361743745	0.468	1.000	1.000	n. s.	0.131	0.205
LL6	73.5	5.5	73.0	4.2	72.7	5.5	74.6	6.5	-1.9	-0.3	1.6	0.457217331	0.751	1.000	0.979	n. s.	0.153	0.268
LR1	87.1	5.6	85.9	5.8	86.0	6.7	89.1	3.6	-3.1	0.1	3.2	**0.007066933**	0.078	1.000	**0.008**	significant	0.269	0.691
LR2	89.4	6.2	89.1	5.6	87.9	8.0	90.9	4.7	-3	-1.2	1.8	0.083101455	0.107	1.000	0.289	n. s.	0.199	0.426
LR3	85.4	6.1	85.2	5.6	83.5	7.0	87.3	5.2	-3.8	-1.7	2.1	**0.050392521**	**0.045**	0.843	0.511	n. s.	0.254	0.637
LR4	84.4	6.1	84.4	6.2	83.2	7.1	85.3	5.2	-2.1	-1.2	0.9	0.492654853	0.708	1.000	1.000	n. s.	0.140	0.229
LR5	77.4	6.3	76.8	5.8	76.5	6.1	78.7	6.9	-2.2	-0.3	1.9	0.266594342	0.648	1.000	0.388	n. s.	0.156	0.278
LR6	72.1	6.5	71.7	6.9	70.3	5.4	73.9	6.7	-3.6	-1.4	2.2	0.132229283	0.177	1.000	0.395	n. s.	0.228	0.538
U1	88.5	2.7	88.2	2.6	89.0	2.4	88.3	2.7	0.7	0.8	0.1	0.186142443	0.275	0.406	1.000	n. s.	0.129	0.200
U2	94.0	2.8	93.6	3.7	95.4	3.5	93.4	3.2	2	1.8	-0.2	**0.021388623**	**0.028**	0.085	1.000	n. s.	0.315	*0.828*
U3	90.7	2.0	90.6	4.7	92.4	4.0	89.7	3.8	2.7	1.8	-0.9	**0.050227058**	**0.044**	0.535	0.810	n. s.	0.555	*1.000*
U4	92.2	2.0	92.5	6.6	93.3	5.2	91.3	4.8	2	0.8	-1.2	0.237385397	0.327	1.000	0.628	n. s.	0.411	*0.971*
U5	95.7	2.2	95.2	7.1	96.9	5.3	95.1	5.3	1.8	1.7	-0.1	0.404431096	0.541	1.000	1.000	n. s.	0.368	*0.930*
U6	98.7	2.2	99.4	7.9	99.4	5.4	97.4	6.7	2	0	-2	0.24488542	0.490	1.000	0.409	n. s.	0.435	*0.984*
L1	89.9	4.1	89.8	2.1	89.5	2.4	90.3	1.9	-0.8	-0.3	0.5	0.360703549	0.919	1.000	0.511	n. s.	0.081	0.104
L2	92.4	4.2	92.7	3.1	91.6	5.1	92.8	3.3	-1.2	-1.1	0.1	0.645576124	1.000	1.000	1.000	n. s.	0.127	0.195
L3	87.3	3.0	87.7	4.8	85.9	5.5	88.7	4.4	-2.8	-1.8	1	0.166188335	0.188	0.595	1.000	n. s.	0.382	*0.947*
L4	85.4	2.5	85.7	5.1	84.4	6.0	86.2	4.8	-1.8	-1.3	0.5	0.499662292	0.791	1.000	1.000	n. s.	0.299	0.786
L5	77.6	2.1	77.4	4.6	76.7	5.7	78.8	6.4	-2.1	-0.7	1.4	0.353692048	0.542	1.000	0.805	n. s.	0.417	*0.975*
L6	73.0	2.4	72.4	4.8	71.5	4.6	74.2	6.0	-2.7	-0.9	1.8	0.11481891	0.180	1.000	0.277	n. s.	0.470	*0.994*

MV = mean value; SD = standard deviation; bold letters = significant p-values ($p<0.05$); n. s. = not significant; italic letters = power (>0.80)

*p-values of pairwise comparisons after Bonferroni-correction as implemented in SPSS Version 29

Supplementary Table S5: Results of the dental cast analysis considering tip of the clinical crown regarding all teeth of the total study population and a statistical comparison (Kruskal-Wallis test) between the different skeletal classes (I, II, III).

Tooth	Study population		Skeletal classes/groups									Statistical tests						
	Total		class I		class II		class III		Differences (MV)			Kruskal-Wallis test (total n=107)				Bonferroni-Hochberg-correction	Effect size *f*	Power
	MV	SD	MV	SD	MV	SD	MV	SD	cII-III	cII-I	cIII-I	*p*-value	cII-III*	cII-I*	cIII-I*	$p_{BH} \leq 0.009083597$		
UR1	3.5	3.8	3.9	3.5	3.1	4.3	3.4	3.8	-0.3	-0.8	-0.5	0.433968731	1.000	0.625	1.000	n. s.	0.086	0.112
UR2	7.0	5.1	7.1	5.9	6.0	5.3	7.7	4.1	-1.7	-1.1	0.6	0.67702477	1.000	1.000	1.000	n. s.	0.137	0.220
UR3	8.8	6.5	9.3	6.9	7.9	5.4	9.1	7.1	-1.2	-1.4	-0.2	0.500242156	0.995	0.843	1.000	n. s.	0.093	0.124
UR4	4.3	5.4	3.6	5.8	4.1	5.2	5.0	5.1	-0.9	0.5	1.4	0.572102168	0.933	1.000	1.000	n. s.	0.109	0.155
UR5	4.3	5.3	3.9	5.2	4.6	5.6	4.4	5.2	0.2	0.7	0.5	0.844762012	1.000	1.000	1.000	n. s.	0.055	0.075
UR6	1.4	7.4	0.4	7.7	3.9	7.8	0.3	6.3	3.6	3.5	-0.1	0.126083454	0.135	1.000	0.619	n. s.	0.222	0.513
UL1	3.2	3.8	3.6	4.1	3.1	3.8	2.9	3.4	0.2	-0.5	-0.7	0.929772849	1.000	1.000	1.000	n. s.	0.078	0.101
UL2	8.5	5.5	9.2	6.4	6.8	4.4	9.1	5.3	-2.3	-2.4	-0.1	0.124407676	0.232	0.214	1.000	n. s.	0.197	0.420
UL3	7.4	7.4	6.5	7.3	6.7	6.1	8.9	8.3	-2.2	0.2	2.4	0.455452793	0.972	1.000	0.750	n. s.	0.149	0.256
UL4	2.5	5.9	1.8	5.9	2.7	6.3	3.0	5.8	-0.3	0.9	1.2	0.556024097	1.000	1.000	1.000	n. s.	0.087	0.115
UL5	1.1	5.7	0.1	6.2	2.2	4.8	1.1	5.9	1.1	2.1	1.0	0.556510091	1.000	0..879	1.000	n. s.	0.148	0.252
UL6	-2.4	8.3	-3.5	8.7	0.5	8.2	-3.6	7.6	4.1	4.0	-0.1	0.162858235	0.221	0.399	1.000	n. s.	0.226	0.527
LL1	-0.4	3.5	-1.1	3.0	1.2	3.9	-1.1	3.1	2.3	2.3	0.0	**0.034532857**	0.149	**0.040**	1.000	n. s.	0.303	0.797
LL2	-1.2	4.0	-1.1	3.3	0.0	4.1	-2.3	4.3	2.3	1.1	-1.2	0.118141212	0.118	1.000	0.775	n. s.	0.234	0.561
LL3	2.3	6.1	2.8	6.3	4.0	4.6	0.4	6.7	3.6	1.2	-2.4	**0.027902462**	**0.024**	0.784	0.362	n. s.	0.246	0.607
LL4	2.7	5.2	2.7	4.5	3.9	5.2	1.9	5.7	2.0	1.2	-0.8	0.230563356	0.262	0.950	1.000	n. s.	0.157	0.279
LL5	9.9	6.1	9.7	5.4	9.6	5.3	10.2	7.5	-0.6	-0.1	0.5	0.698066729	1.000	1.000	1.000	n. s.	0.043	0.065
LL6	9.9	9.1	11.0	9.2	9.7	7.6	9.0	10.2	0.7	-1.3	-2.0	0.450024706	1.000	1.000	0.695	n. s.	0.092	0.123
LR1	-0.7	3.0	-0.3	2.8	-1.0	3.5	-0.7	2.7	-0.3	-0.7	-0.4	0.740949543	1.000	1.000	1.000	n. s.	0.094	0.126
LR2	-1.7	4.3	-1.8	4.8	-1.5	4.1	-1.8	4.0	0.3	0.3	0.0	0.649385212	1.000	1.000	1.000	n. s.	0.032	0.058
LR3	1.0	7.3	1.0	8.6	3.1	5.6	-0.5	6.9	3.6	2.1	-1.5	**0.032362462**	**0.027**	0.660	0.483	n. s.	0.173	0.332
LR4	0.0	5.5	-1.8	6.6	1.9	3.3	0.3	5.4	1.6	3.7	2.1	**0.01486717**	0.368	**0.011**	0.461	n. s.	0.271	0.697
LR5	7.1	6.8	7.8	6.4	6.2	5.7	7.1	7.9	-0.9	-1.6	-0.7	0.440560613	1.000	0.638	1.000	n. s.	0.095	0.126
LR6	8.2	8.1	7.8	8.2	10.2	5.9	6.9	9.3	3.3	2.4	-0.9	0.347160345	0.664	0.565	1.000	n. s.	0.170	0.321
U1	3.3	3.0	3.7	3.2	3.1	2.9	3.2	2.8	-0.1	-0.6	-0.5	0.655833863	1.000	1.000	1.000	n. s.	0.087	0.114
U2	7.8	4.4	8.2	5.3	6.4	4.0	8.4	3.9	-2.0	-1.8	0.2	0.210120921	0.447	0.311	1.000	n. s.	0.201	0.432
U3	8.3	5.9	7.9	6.7	7.3	5.0	9.0	6.1	-1.7	-0.6	1.1	0.530292975	0.782	1.000	1.000	n. s.	0.120	0.178
U4	3.7	5.1	2.7	5.1	3.4	5.0	4.0	4.7	-0.6	0.7	1.3	0.653488207	1.000	1.000	1.000	n. s.	0.106	0.148
U5	2.9	4.8	2.0	4.8	3.4	4.3	2.8	5.0	0.6	1.4	0.8	0.656165286	1.000	1.000	1.000	n. s.	0.118	0.173
U6	-0.6	6.1	-1.5	6.2	2.2	5.8	-1.6	5.8	3.8	3.7	-0.1	0.020440353	**0.021**	0.124	1.000	n. s.	0.284	0.739
L1	-0.5	2.0	-0.7	1.8	0.1	2.5	-0.9	1.8	1.0	0.8	-0.2	0.136902514	0.210	0.288	1.000	n. s.	0.213	0.478
L2	-1.4	3.3	-1.5	3.1	-0.7	3.6	-2.1	3.4	1.4	0.8	-0.6	0.229285579	0.259	1.000	1.000	n. s.	0.184	0.371
L3	1.7	5.9	1.9	6.7	3.5	4.3	0.0	6.0	3.5	1.6	-1.9	**0.009083597**	**0.008**	0.893	0.140	significant	0.242	0.591
L4	1.6	4.8	0.4	4.9	2.9	3.7	1.1	5.0	1.8	2.5	0.7	0.073503538	0.203	0.099	1.000	n. s.	0.215	0.486
L5	8.4	5.2	8.8	4.9	7.9	4.7	8.7	5.9	-0.8	-0.9	-0.1	0.903520184	1.000	1.000	1.000	n. s.	0.076	0.098
L6	9.1	6.3	9.4	6.6	10.0	6.0	7.9	6.4	2.1	0.6	-1.5	0.383403963	0.514	1.000	1.000	n. s.	0.141	0.232

MV = mean value; SD = standard deviation; bold letters = significant p-values ($p<0.05$); n. s. = not significant; italic letters = power (>0.80)

*p-values of pairwise comparisons after Bonferroni-correction as implemented in SPSS Version 29

Supplementary Table S6: Results of the dental cast analysis considering torque of the clinical crown regarding all teeth of the total study population and a statistical comparison (Kruskal-Wallis test) between the different skeletal classes (I, II, III).

Tooth	Study population		Skeletal classes/groups									Statistical tests						
	Total		class I		class II		class III		Differences (MV)			Kruskal-Wallis test				Bonferroni-Holm-correction	Effect size *f*	Power
	MV	SD	MV	SD	MV	SD	MV	SD	cII-III	cII-I	cIII-I	*p*-values	class II-III*	class II-I*	class III-I*	p_{BHC}≤0.001747571		
UR1	5.2	6.7	6.5	5.4	2.2	7.3	6.4	6.6	-4.2	-4.3	-0.1	**0.015206068**	**0.028**	**0.043**	1.000	n. s.	0.293	0.767
UR2	4.2	6.6	5.1	5.6	3.0	7.7	4.5	6.6	-1.5	-2.1	-0.6	0.625091042	1.000	1.000	1.000	n. s.	0.131	0.205
UR3	-7.0	6.4	-6.3	6.9	-7.0	6.0	-7.5	6.3	0.5	-0.7	-1.2	0.756422241	1.000	1.000	1.000	n. s.	0.078	0.101
UR4	-13.1	7.2	-13.4	8.1	-14.5	7.3	-11.6	6.1	-2.9	-1.1	1.8	0.203559401	0.373	1.000	0.379	n. s.	0.166	0.306
UR5	-14.5	7.9	-15.5	8.2	-16.7	6.8	-11.7	7.9	-5.0	-1.2	3.8	**0.023143297**	**0.020**	0.860	0.286	n. s.	0.272	0.699
UR6	-19.9	11.3	-22.5	12.9	-20.8	10.1	-16.6	10.2	-4.2	1.7	5.9	**0.009166391**	**0.039**	1.000	**0.018**	n. s.	0.234	0.521
UL1	5.3	6.7	6.0	5.2	2.6	7.2	6.7	6.9	-4.1	-3.4	0.7	**0.019648206**	**0.018**	0.166	1.000	n. s.	0.263	0.667
UL2	4.5	6.2	5.5	5.7	2.2	7.2	5.5	5.3	-3.3	-3.3	0.0	0.057470058	0.095	0.129	1.000	n. s.	0.246	0.605
UL3	-6.6	7.1	-5.6	7.0	-7.6	7.8	-6.6	6.6	-1.0	-2.0	-1.0	0.726207969	1.000	1.000	1.000	n. s.	0.113	0.162
UL4	-13.3	6.8	-13.5	7.5	-16.1	6.6	-10.9	5.5	-5.2	-2.6	2.6	**0.009137199**	**0.008**	0.864	0.147	n. s.	0.311	*0.817*
UL5	-14.8	7.7	-16.2	8.5	-17.8	6.7	-11.0	6.3	-6.8	-1.6	5.2	**0.000880772**	**0.002**	1.000	**0.009**	significant	0.380	*0.944*
UL6	-20.0	12.1	-21.3	12.9	-21.0	10.1	-18.0	12.9	-3.0	0.3	3.3	0.10316193	0.187	1.000	0.217	n. s.	0.125	0.191
LL1	0.8	7.8	2.7	6.7	4.9	6.2	-4.5	7.2	9.4	2.2	-7.2	**0.00000026377**	**<0.001**	1.000	**<0.001**	significant	0.519	*0.999*
LL2	-2.2	7.0	-0.3	5.6	1.2	6.2	-6.8	6.4	8.0	1.5	-6.5	**0.00000063108**	**<0.001**	1.000	**<0.001**	significant	0.501	*0.997*
LL3	-12.1	6.1	-11.2	5.4	-9.3	5.9	-15.3	5.5	6.0	1.9	-4.1	**0.000016507355703**	**<0.001**	0.513	**0.004**	significant	0.412	*0.972*
LL4	-20.9	5.8	-19.9	4.3	-19.1	6.4	-23.2	5.9	4.1	0.8	-3.3	**0.001747571**	**0.004**	1.000	**0.013**	significant	0.309	*0.812*
LL5	-26.3	6.5	-26.2	6.9	-25.9	5.1	-26.7	7.2	0.8	0.3	-0.5	0.678057031	1.000	1.000	1.000	n. s.	0.051	0.071
LL6	-36.4	5.8	-36.4	5.8	-35.7	4.3	-37.0	7.0	1.3	0.7	-0.6	0.766339829	1.000	1.000	1.000	n. s.	0.091	0.121
LR1	1.2	7.2	3.1	5.4	4.6	6.0	-3.6	7.4	8.2	1.5	-6.7	**0.000001118545384**	**<0.001**	1.000	**<0.001**	significant	0.500	*0.997*
LR2	-2.1	7.0	-1.2	5.3	2.3	6.4	-6.6	6.4	8.9	3.5	-5.4	**0.00000068801**	**<0.001**	0.223	**0.002**	significant	0.524	*0.999*
LR3	-12.5	6.5	-12.2	5.7	-8.9	6.7	-15.7	5.5	6.8	3.3	-3.5	**0.000021006106016**	**<0.001**	0.137	**0.024**	significant	0.426	*0.980*
LR4	-21.1	5.6	-21.3	5.3	-18.8	5.2	-22.9	5.8	4.1	2.5	-1.6	**0.007444022**	**0.006**	0.125	0.876	n. s.	0.299	0.783
LR5	-27.5	6.3	-27.9	5.5	-26.0	4.4	-28.4	7.9	2.4	1.9	-0.5	0.133878272	0.190	0.314	1.000	n. s.	0.162	0.294
LR6	-36.9	5.8	-37.9	6.0	-36.3	5.7	-36.4	5.9	0.1	1.6	1.5	0.469761158	1.000	0.722	1.000	n. s.	0.126	0.193
U1	5.1	6.5	6.2	4.9	2.4	7.1	6.6	6.6	-4.2	-3.8	0.4	**0.014623438**	**0.016**	0.093	1.000	n. s.	0.286	0.745
U2	4.3	5.9	5.3	5.0	2.6	7.1	5.0	5.6	-2.4	-2.7	-0.3	0.272240975	0.491	0.461	1.000	n. s.	0.200	0.431
U3	-6.6	6.0	-6.0	6.6	-7.3	6.3	-7.1	5.5	-0.2	-1.3	-1.1	0.8640163	1.000	1.000	1.000	n. s.	0.095	0.127
U4	-13.0	6.5	-13.4	7.5	-15.3	6.6	-11.3	5.1	-4.0	-1.9	2.1	**0.027632459**	**0.038**	1.000	0.132	n. s.	0.251	0.623
U5	-14.3	7.2	-15.9	7.5	-17.2	6.4	-11.3	6.1	-5.9	-1.3	4.6	**0.001182087**	**0.002**	1.000	**0.018**	significant	0.355	*0.910*
U6	-19.9	11.5	-21.9	12.5	-20.9	9.7	-17.3	11.1	-3.6	1.0	4.6	**0.014947728**	**0.032**	1.000	**0.046**	n. s.	0.175	0.339
L1	0.9	7.4	2.9	5.9	4.8	5.8	-4.1	7.1	8.9	1.9	-7.0	**0.00000034214**	**<0.001**	1.000	**<0.001**	significant	0.522	*0.999*
L2	-2.3	6.8	-0.7	5.2	1.8	6.1	-6.7	6.1	8.5	2.5	-6.0	**0.0000003232**	**<0.001**	0.850	**<0.001**	significant	0.527	*0.999*
L3	-12.2	5.9	-11.7	4.7	-9.1	5.9	-15.5	5.0	6.4	2.6	-3.8	**0.000002478436488**	**<0.001**	0.164	**0.005**	significant	0.446	*0.988*
L4	-20.8	5.1	-20.6	3.7	-19.0	5.2	-23.0	4.9	4.0	1.6	-2.4	**0.000635222**	**<0.001**	0.473	**0.049**	significant	0.326	*0.846*
L5	-26.6	5.9	-27.0	5.3	-26.0	4.2	-27.6	6.9	1.6	1.0	-0.6	0.402847361	0.539	1.000	1.000	n. s.	0.111	0.158
L6	-36.2	5.6	-37.2	5.4	-36.0	4.4	-36.7	5.7	0.7	1.2	0.5	0.720881326	1.000	1.000	1.000	n. s.	0.086	0.113

MV = mean value; SD = standard deviation; bold letters = significant p-values ($p<0.05$); n. s. = not significant; italic letters = power (>0.80)

*p-values of pairwise comparisons after Bonferroni-correction as implemented in SPSS Version 29

Supplementary Table S7: Interpretation of differences regarding measured tip of teeth considering the a) orthopantomogram evaluation of the long axis and b) dental cast analysis of the clinical crown between the different skeletal classes (I, II, III).

		TIP: Skeletal class II vs. skeletal class III				TIP: Skeletal class II vs. skeletal class I				TIP: Skeletal class III vs. skeletal class I			
		Long Axis[1]	Clinical crown[2]	Long Axis[1]	Clinical crown[2]	Long Axis[1]	Clinical crown[2]	Long Axis[1]	Clinical crown[2]	Long Axis[1]	Clinical crown[2]	Long Axis[1]	Clinical crown[2]
		cII-III	cII-III	Interpretation	Interpretation	cII-I	cII-I	Interpretation	Interpretation	cIII-I	cIII-I	Interpretation	Interpretation
UPPER JAW	UR1	-0.4	-0.3	mesial	distal	0.8	-0.8	**distal**	**distal**	1.2	-0.5	**distal**	**distal**
	UR2	0.7	-1.7	**distal**	**distal**	1.1	-1.1	**distal**	**distal**	0.4	0.6	distal	mesial
	UR3	1.1	-1.2	**distal**	**distal**	0.1	-1.4	**distal**	**distal**	-1	-0.2	mesial	distal
	UR4	0.9	-0.9	**distal**	**distal**	-0.2	0.5	**mesial**	**mesial**	-1.1	1.4	**mesial**	**mesial**
	UR5	1.8	0.2	distal	mesial	1	0.7	distal	mesial	-0.8	0.5	**mesial**	**mesial**
	UR6	2.1	3.6	distal	mesial	-1.5	3.5	**mesial**	**mesial**	-3.6	-0.1	mesial	distal
	UL1	2	0.2	distal	mesial	0.9	-0.5	**distal**	**distal**	-1.1	-0.7	mesial	distal
	UL2	3.3	-2.3	**distal**	**distal**	2.7	-2.4	**distal**	**distal**	-0.6	-0.1	mesial	distal
	UL3	4.1	-2.2	**distal**	**distal**	3.4	0.2	distal	mesial	-0.7	2.4	**mesial**	**mesial**
	UL4	3	-0.3	**distal**	**distal**	1.8	0.9	distal	mesial	-1.2	1.2	**mesial**	**mesial**
	UL5	1.9	1.1	distal	mesial	2.3	2.1	distal	mesial	0.4	1	distal	mesial
	UL6	1.9	4.1	distal	mesial	1.5	4	distal	mesial	-0.4	-0.1	mesial	distal
LOWER JAW	LL1	1.5	2.3	distal	mesial	-0.7	2.3	**mesial**	**mesial**	-2.2	0	mesial	n. a.
	LL2	0.6	2.3	distal	mesial	-1	1.1	**mesial**	**mesial**	-1.6	-1.2	mesial	distal
	LL3	-1.7	3.6	**mesial**	**mesial**	-1.7	1.2	**mesial**	**mesial**	0	-2.4	**distal**	**distal**
	LL4	-1.6	2	**mesial**	**mesial**	-1.5	1.2	**mesial**	**mesial**	0.1	-0.8	**distal**	**distal**
	LL5	-2	-0.6	mesial	distal	-1	-0.1	mesial	distal	1	0.5	distal	mesial
	LL6	-1.9	0.7	**mesial**	**mesial**	-0.3	-1.3	mesial	distal	1.6	-2	**distal**	**distal**
	LR1	-3.1	-0.3	mesial	distal	0.1	-0.7	distal	distal	3.2	-0.4	**distal**	**distal**
	LR2	-3	0.3	**mesial**	**mesial**	-1.2	0.3	**mesial**	**mesial**	1.8	0	**distal**	n. a.
	LR3	-3.8	3.6	**mesial**	**mesial**	-1.7	2.1	**mesial**	**mesial**	2.1	-1.5	**distal**	**distal**
	LR4	-2.1	1.6	**mesial**	**mesial**	-1.2	3.7	**mesial**	**mesial**	0.9	2.1	distal	mesial
	LR5	-2.2	-0.9	mesial	distal	-0.3	-1.6	mesial	distal	1.9	-0.7	**distal**	**distal**
	LR6	-3.6	3.3	**mesial**	**mesial**	-1.4	2.4	mesial	mesial	2.2	-0.9	**distal**	**distal**
UPPER JAW	U1	0.7	-0.1	**distal**	**distal**	0.8	-0.6	**distal**	**distal**	0.1	-0.5	**distal**	**distal**
	U2	2	-2	**distal**	**distal**	1.8	-1.8	**distal**	**distal**	-0.2	0.2	**mesial**	**mesial**
	U3	2.7	-1.7	**distal**	**distal**	1.8	-0.6	**distal**	**distal**	-0.9	1.1	**mesial**	**mesial**
	U4	2	-0.6	**distal**	**distal**	0.8	0.7	distal	mesial	-1.2	1.3	**mesial**	**mesial**
	U5	1.8	0.6	distal	mesial	1.7	1.4	distal	mesial	-0.1	0.8	**mesial**	**mesial**
	U6	2	3.8	distal	mesial	0	3.7	n. a.	mesial	-2	-0.1	mesial	distal
LOWER JAW	L1	-0.8	1	**mesial**	**mesial**	-0.3	0.8	**mesial**	**mesial**	0.5	-0.2	**distal**	**distal**
	L2	-1.2	1.4	**mesial**	**mesial**	-1.1	0.8	**mesial**	**mesial**	0.1	-0.6	**distal**	**distal**
	L3	-2.8	3.5	**mesial**	**mesial**	-1.8	1.6	**mesial**	**mesial**	1	-1.9	**distal**	**distal**
	L4	-1.8	1.8	**mesial**	**mesial**	-1.3	2.5	**mesial**	**mesial**	0.5	0.7	distal	mesial
	L5	-2.1	-0.8	mesial	distal	-0.7	-0.9	mesial	distal	1.4	-0.1	**distal**	**distal**
	L6	-2.7	2.1	**mesial**	**mesial**	-0.9	0.6	**mesial**	**mesial**	1.8	-1.5	**distal**	**distal**

[1] a **smaller measured angle** corresponds to a greater extent of **mesial tip**, whereas a **greater angle** indicates a higher amount of **distal tip**

[2] a **greater measured angle** indicates a greater extent of **mesial tip**, while a **smaller angle** corresponds to a greater extent of **distal tip**
